# Phosphorylation Provides a Negative Mode of Regulation for the Yeast Rab GTPase Sec4p

**DOI:** 10.1371/journal.pone.0024332

**Published:** 2011-09-12

**Authors:** Christopher D. Heger, Christiane D. Wrann, Ruth N. Collins

**Affiliations:** 1 Graduate Program in Pharmacology, Cornell University, Ithaca, New York, United States of America; 2 Leadership Program for Veterinary Students, College of Veterinary Medicine, Cornell University, Ithaca, New York, United States of America; 3 Department of Molecular Medicine, Cornell University, Ithaca, New York, United States of America; Institut Européen de Chimie et Biologie, France

## Abstract

The Rab family of Ras-related GTPases are part of a complex signaling circuitry in eukaryotic cells, yet we understand little about the mechanisms that underlie Rab protein participation in such signal transduction networks, or how these networks are integrated at the physiological level. Reversible protein phosphorylation is widely used by cells as a signaling mechanism. Several phospho-Rabs have been identified, however the functional consequences of the modification appear to be diverse and need to be evaluated on an individual basis. In this study we demonstrate a role for phosphorylation as a negative regulatory event for the action of the yeast Rab GTPase Sec4p in regulating polarized growth. Our data suggest that the phosphorylation of the Rab Sec4p prevents interactions with its effector, the exocyst component Sec15p, and that the inhibition may be relieved by a PP2A phosphatase complex containing the regulatory subunit Cdc55p.

## Introduction

Our current view of membrane traffic is far from that of a distribution network with machinery that passively responds to the tasks of cargo collection and transport. Elucidation of the molecular mechanisms that regulate the production and consumption of transport carriers is a significant challenge in cell biology, and is central to understanding the underlying contribution of membrane traffic to topics as diverse as apico-basolateral polarity and cell proliferation, how organelles can be manipulated by pathogens to facilitate intracellular entry and residence, and the modulation of cellular morphology during development to generate cell-type specificity. Each stage of transport is expected to be under the control of a variety of different signals requiring regulatory checks and balances and coordination with other cellular events.

The Rab family of GTPases are central players in the regulation of membrane traffic. Rab GTPases exert control by harnessing the conformational changes associated with GTP binding and hydrolysis to a cycle of transition between membranes and cytosol. At least one or more Rab family members are a necessary component of all transport steps in the secretory and endocytic systems. Because phosphorylation is a general regulatory modification utilized by diverse signal transduction pathways, its presence on Rab proteins represents a possible intervention point to understand the mechanisms by which membrane traffic is coordinated with other cellular pathways. Several global studies of the yeast phosphoproteome have been performed, identifying sites of phosphorylation on three Rab proteins, Sec4p, Ypt1p and Vps21p [1], [2], [3], [4]. In particular, the Sec4p GTPase has been identified as a multi-site phosphoprotein by two independent studies [2], [5]. Sec4p contains 4–5 serine phosphorylation sites situated within two stretches close to the NH_2_- and COOH-termini, ^6^TVpSASpSGNGK^15^, and ^196^EGNIpSINpSGSGNS^209^. In this study we have investigated the consequence(s) of this modification for Sec4p function.

Sec4p is encoded by an essential gene and is a critical mediator for the pathway that delivers post-Golgi vesicles to the plasma membrane [6]. This trafficking step is spatially and temporally regulated to the sites of active growth. Reflecting this fact, Sec4p is found at the tip of newly growing cells and at the neck region between dividing cells [7]. The closest mammalian orthologs of Sec4p are Rab8 and Rab13 [8], and these proteins also regulate post-Golgi trafficking pathways. The consequences of Rab GTPase activation are transmitted to downstream effectors, proteins or protein complexes that bind to the nucleotide-activated or GTP-bound conformation of a specific Rab protein [9]. Several effectors have been identified for Sec4p including Sec15p, a member of the octameric exocyst complex [10]. The exocyst complex is also an effector for other Ras-related small GTPases, and is known to be a central player that serves as an intersection point for multiple signal transduction pathways [11], [12], [13], [14], [15], [16], [17], [18], [19], [20]. The outcome of our study suggests that Sec4p phosphorylation may act in a negative regulatory capacity, disconnecting Sec4p from Sec15p engagement and providing a route for the cell to control exocyst function.

## Methods

### Yeast Strains, Media and Reagents

The *Saccharomyces cerevisiae* strains used in this study are listed in Table S1 and were created using standard manipulations. For Western blot analysis, gels were transferred to PVDF Immobilon membrane (Millipore) for 2 hrs prior to probing with antibodies including anti-GFP (Chemicon) and anti-Sec26p [21]. Blots were subsequently incubated with the appropriate secondary antibody coupled to alkaline phosphatase and imaged using CDP-Star chemiluminescence reagent (Perkin Elmer) and recorded with Fuji LAS3000. We examined synthetic interactions of *cdc55Δ* or *rts1Δ* with *sec4-8* by deleting these genes in a diploid *sec4-8/sec4-8* strain that was subsequently sporulated to haploids. Replacement of the endogenous copy of SEC4 was performed with a plasmid shuffle assay outlined in Figure S4.

### Fluorescence Microscopy

Live cells were analyzed with a Nikon Eclipse E600 microscope, 100× (1.4NA) lens, 1× optivar (0.08 µm/pixel), and imaged using a Sensicam EM High Performance camera (The Cooke Corporation). Differential interference contrast (DIC) images were taken in a single plane. Fluorescent images were collected as a z-series with a 0.2 µm z step size, generally with 20–28 slices per stack. Images were captured with IP Lab 3.6.5 software and deconvolution performed with Autodeblur and Autovisualize 9.1 software.

### Yeast Two-Hybrid Assays

The gene sequences encoding *SEC4* and alleles of *SEC4* were inserted into the bait two-hybrid vector pAS2-1 with BamHI/EcoRI. For prey constructs, the gene encoding regions of *GDI1* and *SEC15* were ligated into pACT2 with BamHI/Xho1. Plasmids were co-transformed into Y190 and allowed to grow for several days before processing for β-galactosidase activity. Due to batch variability in yeast two-hybrid (Y2H) assays each complete experiment was carried out in a set that included positive and negative controls. We also commonly observed variability in the Y2H system between two otherwise identical constructs and so two independently generated constructs were used to confirm interactions observed in our experiments. Pairs of plasmids were cotransformed into the yeast strain and at least 30 independent colonies were assayed for β-galactosidase activity. β-galactosidase activity was determined with the chromogenic substrate X-gal.

### Electron Microscopy

For electron microscopy, samples were taken from yeast cultures grown at different temperatures as indicated in the legend to Fig. 2. Fixation was achieved by washing 50 OD_600_ units of cells (1 to 2 OD_600_ units/ml) once with distilled water and then resuspending the cells in 10 ml of fresh 2% KMnO_4_ and incubating them for 2 h at room temperature. Fixed cells were washed twice with distilled water, dehydrated in a graded series of ethanol washes, and embedded in SPURR resin (Electron Microscopy Sciences). Silver to pale-gold sections (about 60 nm) were contrasted with uranyl acetate and lead citrate for 5 min each. Sections were examined in a FEI Philips TECHNAI 12 BioTwin electron microscope at 100 kV or 80 kV.

## Results

### Role of Sec4p phosphorylation sites

To experimentally determine the functional relevance of the Sec4p phosphorylation sites, we replaced these serine residues with the phosphomimetic residues glutamic acid or aspartic acid (E or D, [22]), or with nonphosphorylatable alanines (A). To assay the functionality of these constructs, we asked if they could replace the endogenous copy of *SEC4*. As *SEC4* is an essential gene we made use of a plasmid shuffle system with the counter-selectable *URA3* marker, to generate cells whose only source of the essential gene *SEC4* was the novel construct provided [23]. Briefly, the genomic copy of SEC4 is deleted and the cell viability maintained with an episomal copy of wild type SEC4 in a plasmid containing *URA3*. After transformation with an alternative plasmid containing the novel *sec4* allele, the cells are plated on 5FOA to select against the *URA3*-containing plasmid (Supplementary Data), leaving cells with the novel *sec4* allele as the only cellular source of Sec4p function.

**Figure 1:**
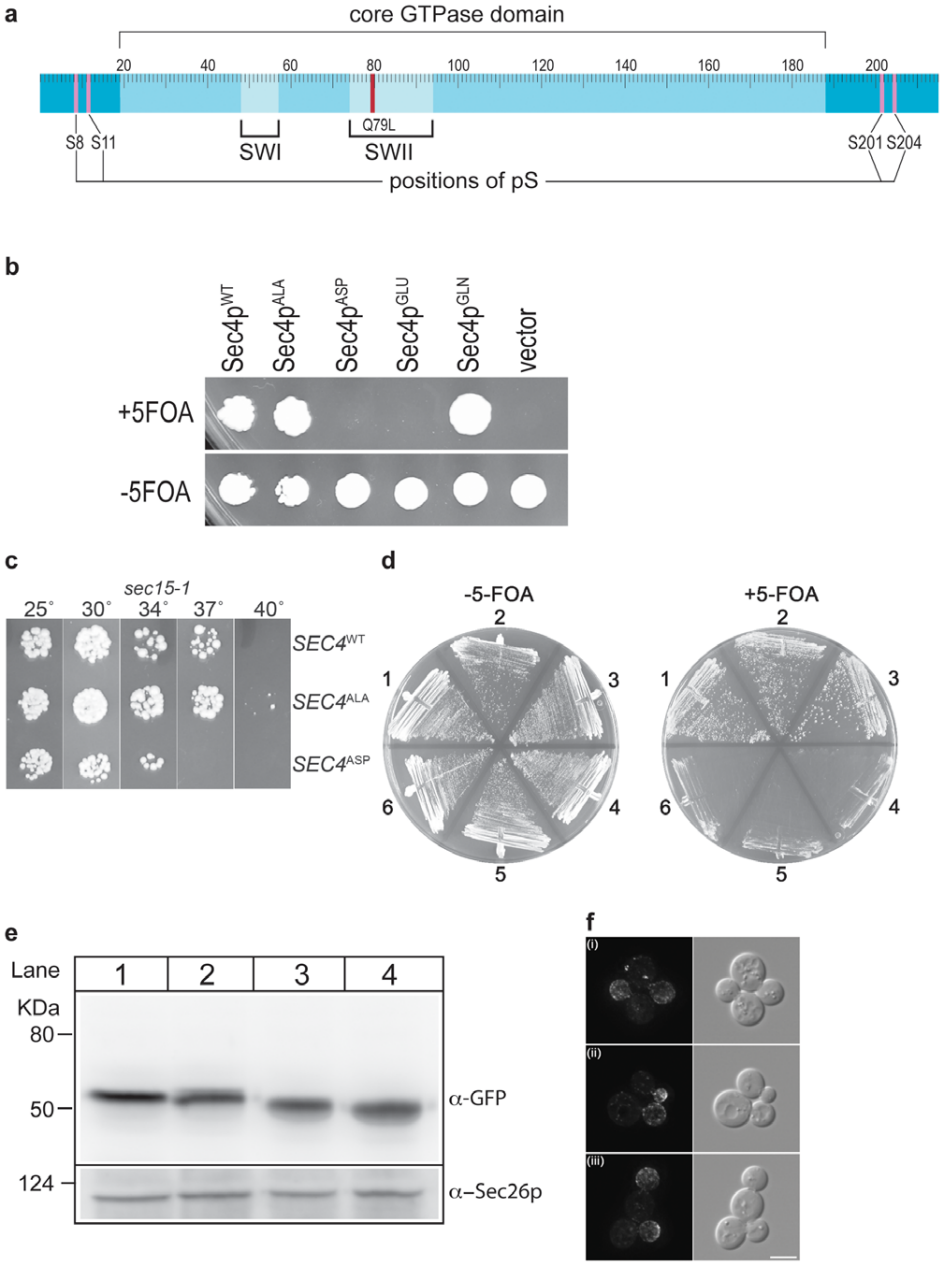
Mutation of Sec4p phosphorylation sites. **a.** Schematic of Sec4p. The positions of the nucleotide-dependent conformational switches (SWI, SWII) and the catalytic Q79 residue are shown relative to the positions of the phosphorylated serines (S8, S11, S201, S204). **b.** Functionality of GFP-tagged Sec4p phosphorylation mutants. Sec4p phosphorylation sites were mutated as described in the text to alanine (Sec4p^ALA^; ^8^AAAA^11^ ^201^AINA^204^), aspartic acid (Sec4p^ASP^; ^8^DADD^11^ ^201^DIND^204^), glutamic acid (Sec4p^GLU^;^ 8^EAEE^11^ ^201^EINE^204^) or glutamine (Sec4p^GLN^; ^8^QAQQ^11^ ^201^QINQ^204^) residues and transformed into the tester strain along with wild type Sec4p (Sec4p^WT^; ^8^SASS^11^ ^201^SINS^204^) and vector (pRS315) as positive and negative controls, respectively. Transformants were spotted onto media with (+) or without (−) 5-Fluoroorotic acid (5-FOA) and incubated at 30°C for 3 days. **c.** Expression of Sec4p mutants without NH_2_-terminal tag. Cells containing episomal plasmids with the indicated *SEC4* constructs as the sole cellular source of *SEC4* were tested for growth ability in the sensitized background of *sec15-1*. **d.** Sec4p Q79L phosphomimetics are non-functional in *sec15-1* cells. Constructs of Sec4p^ALA^Q79L (1), Sec4p Q79L (2), GFP-Sec4p (3), vector (4), Sec4p^GLU^Q79L (5) and Sec4p^ASP^Q79L (6) were transformed into *sec15-1 SEC4Δ* cells. Resulting transformants were struck onto media with (+) or without (−) 5-FOA and incubated at 25°C for 3 days. **e.** Expression of GFP-Sec4p phosphomutants. Cells containing GFP-tagged Sec4p phosphomutants were grown to mid-log phase prior to harvesting. Clarified supernatants of yeast extracts were generated by glass bead lysis, and subjected to SDS-PAGE and Western blot analysis with α-GFP. α-Sec26p was used separately as a lysate loading control. Lane 1 Sec4p^GLU^, Lane 2 Sec4p^ASP^, Lane 3 Sec4p^WT^, Lane 4 Sec4p^ALA^. **f.** Localization of GFP-tagged Sec4p phosphomutants. (i) GFP-Sec4p^WT^ (RCY3122), (ii) GFP-Sec4p^ASP^ (RCY3124) (iii) GFP-Sec4p^ALA^ (RCY3126) cells were examined by fluorescence microscopy. The Sec4p^WT^ and Sec4p^ALA^ constructs were expressed as the sole copy of *SEC4*. Bar = 5 µm.

In our analysis, we also included the serine at position 10 reasoning that its proximity to the phosphorylated residues of S8 and S11 might allow it to be utilized as a bypass phosphorylation site in the case of relaxed positional preference (Figure 1A). In addition, the inclusion of S10 may compensate for amino acid substitutions that are poorly phosphomimetic. Combined mutagenesis of the phosphorylated serine residues at all five positions revealed that an alanine replacement (Sec4p^ALA^) retained in vivo functionality. However, replacement of the serine residues with either of the phosphomimetic amino acids aspartic acid (Sec4p^ASP^) or glutamic acid (Sec4p^GLU^) resulted in an allele that was unable to functionally complement the wild type gene (Figure 1B). Substitution with glutamine (Sec4p^GLN^), a neutral polar amino acid of the same size as the phosphomimetic amino acids, had no effect on functionality indicative of the suggestion that aspartic and glutamic acid residues are serving as phosphomimetics. These data indicate that the phosphorylation of serines at position S8, S11, S201, S204 is not required for Sec4p functionality. Rather, they suggest that Sec4p is phosphorylated in vivo as part of a cycle where the phosphorylated state is inhibitory and the impact of phosphomimetic substitutions are that they lock Sec4p in an inhibitory state. These experiments used a GFP-tagged version of Sec4p, however the effect of the different mutants was unaffected by the nature of the GFP moiety as an alternative tag, maltose binding protein (MBP), had the same effect (Supplementary Data, Figure S1).

We also used an alternative method to check the functionality of untagged *SEC4* constructs in vivo. This method exploits the fact that a duplication of the *SEC4* gene can suppress the exocytic mutant *sec15-1*, a component of the exocyst that is an effector for the Sec4p GTPase [24]. In this experiment, shown in Fig. 1C, we used untagged versions of the wild type and Sec4p mutant constructs with a similar result, that alanine substitutions gave robust function and the aspartic acid substitutions abrogated the function of the protein. The alanine mutant showed a slight advantage over wild type in that it was able to weakly suppress at 40°C. We also examined untagged versions of these *sec4* alleles incorporated into the GTP-hydrolysis deficient Q79L mutant of Sec4p [25], which yielded the same result, namely that a construct where the phosphorylated serine residues were substituted with phosphomimetics was non-functional, relative to the substitutions with alanine (Fig. 1D). Thus a situation that compromises Sec4p function, whether artificially with NH_2_-terminal tagging, or in vivo using a *sec15* mutant or a GTP-hydrolysis deficient form of Sec4p, allows us to discriminate between levels of Sec4p action. Overall, we conclude that alanine substitutions at S8, S10, S11, S201 and S204 has the same or slightly enhanced functionality relative to wild type Sec4p, and the aspartic acid substitutions at these positions give rise to a severely disabled and non-functional protein. Assuming that the aspartic or glutamic acid substitutions at the positions of the phosphorylated serines represent a phosphomimetic version of the wild type Sec4p, these data suggest that phosphorylation is detrimental to the function of Sec4p.

To understand the loss of function of phosphorylated Sec4p we first considered the possibility that the resulting protein was expressed at levels too low to sustain viability, or that the protein is incorrectly localized, a known prerequisite for Rab protein functionality. Western blots of total cellular lysates demonstrated that the level of the non-functional phosphomimetic Sec4p was comparable to wild type Sec4p (Fig. 1E) and had no apparent effect on protein stability. Examination of GFP-tagged versions of the mutated serines showed no deficiencies in Sec4p localization (Fig. 1F). We then examined the possibility that phosphomimetic Sec4p might have an altered nucleotide binding or hydrolysis cycle. Substitution of serines 8, 10, 11, 201, 204 with either alanine or aspartic acid also did not affect its intrinsic ability to bind and hydrolyze guanine nucleotides (Supplementary Data, Figure S2A).

### Sec4p phosphorylation blocks interactions with the exocyst component Sec15p

What are the consequences of phosphorylation for Sec4p function? The fact that substitution of phosphorylated serine residues with glutamic or aspartic acid residues cannot provide function (Fig. 1B) suggests that phosphorylation blocks an action of Sec4p that is critical for cell viability. As substitution of serines 8, 10, 11, 201, 204 with either alanine or aspartic acid residues had no effect on the intrinsic ability of the Rab GTPase to bind and hydrolyze guanine nucleotides (Supplementary Data), we next examined the ability of phosphorylation to influence the action of the regulators of Sec4p. We examined the known Sec4p activators, Dss4p and Sec2p on their ability to regulate recombinant phosphomimetic (serine to aspartic acid) or phosphoablated (serine to alanine) versions of Sec4p. In this series of experiments, exchange assays were performed to examine the ability of Dss4p or Sec2p to influence the rate of GDP/GTP exchange on Sec4p, however we saw no effect between the different Sec4p alleles (Supplementary Data, Figure S2B). Similarly, the GTPase activating protein for Sec4p, Gyp1p did not discriminate between wild type, phosphomimetic or phosphoablated versions of Sec4p.

As phosphomimetic substitutions did not appear to affect the ability of Sec4p to undergo a normal nucleotide cycle, we hypothesized that phosphorylation might impact the ability of Sec4p to act in concert with its effectors. Downstream of Sec4p activation is the action of the SNARE protein Sec9p via two known effectors, Sro7p [26] and the exocyst component Sec15p [10]. Of these known effectors, only the action of Sec15p is essential to support cell viability. We tested the interaction between phosphomimetic Sec4p and Sec15p with the two-hybrid assay previously used to demonstrate effector interactions between Sec4p and Sec15p [10]. The results are shown in Table 1. Wild type Sec4p and Sec4p^ALA^ showed interactions with Sec15p. In contrast, Sec15p interactions were abolished with Sec4p^ASP^. To further investigate interactions with Sec15p, we made use of the GTP hydrolysis defective allele of Sec4p [25], which has been shown to have an enhanced interaction with Sec15p [10]. The Q79L point mutation of Sec4p (Sec4pQ79L) stimulated interaction with Sec15p, as did Sec4p^ALA^Q79L and these interactions were absent with Sec4p^ASP^Q79L. As previously demonstrated, Sec15p interactions required the effector domain of Sec4p; a Q79L construct where the Switch I loop was replaced with equivalent residues from Ypt1p, Sec4 ^EF YPT1^Q79L, did not interact with Sec15p [10]. Rab- GDP-Displacement inhibitor (Rab-GDI), a universal Rab GTPase regulator, that extracts all Rab proteins from membranes [27], [28], showed equivalent interactions with all Sec4p mutants tested. Sec15p interactions were abolished when the NH_2_-terminal serine residues were replaced by phosphomimetics (D_8_D_10_D_11_ and GTP-hydrolysis deficient). Sec15p interactions were also abolished when the core GTPase domain was trimmed of its NH_2_-terminal extension (Sec4p Δ 1–18) while this construct showed robust interaction with Rab-GDI. These data show that in addition to the nucleotide-dependency and previously identified effector region [10], the interaction between Sec15p and Sec4p requires peptide sequences that protrude beyond the core GTPase domain. Phosphomimetic substitutions of the phosphorylated serines in these flexible extensions blocks Sec15p interaction suggesting that phosphorylation of Sec4p is deployed in a negative regulatory mode to eliminate exocyst engagement that is crucial for successful exocytosis [29].

**Table 1:** Sec15p interactions with Sec4p are abbrogated by phosphomimetics.

Bait construct	Description	Prey construct	Result
Sec4p^WT^	Wild type	SEC15	++
Sec4p^ALA^	^8^AAAA^11^ ^201^AINA^204^	SEC15	++
Sec4p^ASP^	^8^DADD^11^ ^201^DIND^204^	SEC15	−
Sec4p Q79L	GTP-hydrolysis deficient	SEC15	++++
Sec4p^EF YPT1^Q79L	GTP-hydrolysis deficient, YPT1 SWI domain	SEC15	−
Sec4p^ALA^Q79L	^8^AAAA^11^ ^201^AINA^204^ and GTP-hydrolysis deficient	SEC15	++++
Sec4p^ASP^Q79L	^8^DADD^11^ ^201^DIND^204^ and GTP-hydrolysis deficient	SEC15	−
Sec4p ^8^AASA^11^ ^201^DIND^204^ Q79L	^8^AASA^11^ ^201^DIND^204^ and GTP-hydrolysis deficient	SEC15	++
Sec4p NH_2_-terminal ^ASP^Q79L	^8^DADD^11^ ^201^SINS^204^ and GTP-hydrolysis deficient	SEC15	−
Sec4p^ΔN18^Q79L	GTP-hydrolysis deficient, ΔN1-18	SEC15	−
Sec4p ^8^DASD^11^ ^201^DIND^204^ Q79L	^8^DASD^11^ ^201^DIND^204^ and GTP-hydrolysis deficient	SEC15	−
Sec4p^WT^	Wild type	GDI1	++++
Sec4p^ALA^	^8^AAAA^11^ ^201^AINA^204^	GDI1	++++
Sec4p^ASP^	^8^DADD^11^ ^201^DIND^204^	GDI1	+++
Sec4p Q79L	GTP-hydrolysis deficient	GDI1	++++
Sec4p^EF YPT1^Q79L	GTP-hydrolysis deficient, YPT1 SWI domain	GDI1	++++
Sec4p^ALA^Q79L	^8^AAAA^11^ ^201^AINA^204^ and GTP-hydrolysis deficient	GDI1	++++
Sec4p^ASP^Q79L	^8^DADD^11^ ^201^DIND^204^ and GTP-hydrolysis deficient	GDI1	+++
Sec4p NH_2_-terminal ^ASP^Q79L	^8^DADD^11^ ^201^SINS^204^ and GTP-hydrolysis deficient	GDI1	++++
Sec4p^ΔN18^Q79L	GTP-hydrolysis deficient, ΔN1-18	GDI1	++++
Sec4p ^8^DASD^11^ ^201^DIND^204^ Q79L	^8^DASD^11^ ^201^DIND^204^ and GTP-hydrolysis deficient	GDI1	++++
Sec4p^WT^	Wild type	vector	−
Sec4p^ALA^	^8^AAAA^11^ ^201^AINA^204^	vector	−
Sec4p^ASP^	^8^DADD^11^ ^201^DIND^204^	vector	−
Sec4p Q79L	GTP-hydrolysis deficient	vector	−
Sec4p^ΔN18^Q79L	GTP-hydrolysis deficient, ΔN1-18	vector	−
Sec4p^EF YPT1^Q79L	GTP-hydrolysis deficient, YPT1 SWI domain	vector	−
Sec4p^ALA^Q79L	^8^AAAA^11^ ^201^AINA^204^ and GTP-hydrolysis deficient	vector	−
Sec4p^ASP^Q79L	^8^DADD^11^ ^201^DIND^204^ and GTP-hydrolysis deficient	vector	−
Sec4p NH_2_-terminal ^ASP^Q79L	^8^DADD^11^ ^201^SINS^204^ and GTP-hydrolysis deficient	vector	−
Sec4p^ΔN18^Q79L	GTP-hydrolysis deficient, ΔN1-18	vector	−
Sec4p ^8^DASD^11^ ^201^DIND^204^ Q79L	^8^DASD^11^ ^201^DIND^204^ and GTP-hydrolysis deficient	vector	−

The interaction between Sec15p, Gdi1p and Sec4p was assessed using the yeast two-hybrid system. Reporter cells were cotransformed with a prey vector encoding *SEC15* and a bait plasmid encoding either wild type or GTPase deficient mutant Sec4p Q79L with phosphorylated serine residues replaced with either alanine or aspartic acid. The strength of the interaction was determined using a β-galactosidase reporter assay and assigned a relative strength of (++++, strong interaction to −, no interaction).

### Mutational analysis of Sec4p phosphorylation sites

**Figure 2:**
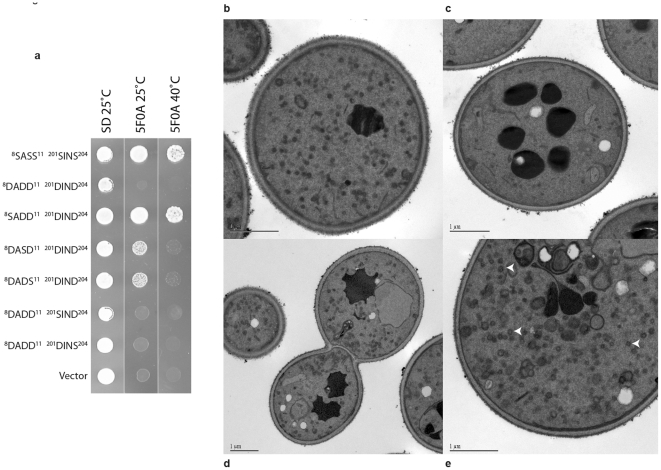
Mutational analysis of phosphorylated serine residues. **a.** Functional analysis of cells containing *sec4* alleles that differ in the substitution of residues at the sites of serine phosphorylation as indicated. Each of the Sec4p mutant constructs was transformed into *SEC4*Δ along with a vector control (pRS315) and plated on SD media for 3d prior to streaking on either SD or SCD+5-FOA at the indicated temperatures to assess functionality. **b–e.** Thin section electron microscopy of *sec4* phosphomimetic alleles. Cells containing phosphomimetic *sec4* alleles *sec4* ^8^DASD^11^ ^201^DIND^204^ (b) and *sec4* ^8^DADS^11^ ^201^DIND^204^ (d,e), and an isogenic wild type control (c), were examined by thin-section electron microscopy. Representative examples of each strain are shown. Bar = 1 µm. White arrows indicate examples of accumulated vesicles at the restrictive temperature in the *sec4* phosphomimetic alleles.

To understand the effects of mutations at the individual sites of the phosphorylated serines, we then examined the effect of replacing each mutated residue in the phosphomimetic substituted protein (Sec4p^ASP^; ^8^DADD^11^ ^201^DIND^204^) back to the wild type serine to determine if there were individual contributions that could be analyzed for each phosphorylated serine residue. These data are shown in Figure 2a. Restoration of serine at the NH_2_-terminus at position 8 (^8^SADD^11^ ^201^DIND^204^) restored viability to the complete phosphomimetic construct. Replacement with serine at position 10 or 11 also restored viability, but the cells bearing these constructs showed a thermosensitive phenotype. Reintroduction of serine residues in either of the two COOH-terminal positions did not alter the inability of the construct to provide Sec4p function.

To examine the phenotype of the thermosensitive sec4 alleles directly, we made use of thin-section electron microscopy. Cells with *sec4* ^8^DASD^11^ ^201^DIND^204^ and *sec4* ^8^DADS^11^ ^201^DIND^204^ were grown at the permissive temperature and shifted for 1 h to restrictive temperature before being fixed and prepared for electron microscopy (Fig. 2b–e). The *sec4* phosphomimetic mutant cells (Fig. 2 b,d,e) showed an accumulation of 80–100 nm secretory vesicles while vesicle accumulation in the isogenic wild type controls was absent (Fig. 2c). The vesicle accumulation phenotype is a diagnostic feature of all post-Golgi secretory mutants, including *sec4-8*, a mutant hypomorph resulting from protein instability.

Together, these data suggest that the impact of the phosphorylated serines could be context dependent, perhaps indicating a requirement for a hierarchical set of kinases to fine-tune the regulatory signal. Overall, it appears that the NH_2_-terminal residues provide a major contribution to the non-functional Sec4p^ASP^. This is consistent with the requirement of this region for productive interaction with Sec15p. The COOH-terminal residues 201 and 204 can also make a contribution to the overall functional effect of phosphorylation. The structural considerations suggesting such a possibility and the implications for the biological regulation of Sec4p function are further explored in the discussion.

### Influence of phosphatase activity

Our data suggested that the reduced functional impact when the phosphorylatable serines were replaced with aspartic or glutamic acid residues could indicate a negative regulatory role for phosphorylation. In situations where phosphorylation is not necessary for function but the activity of the phosphorylated protein is blocked, one expectation is that the system must rely on a critical phosphatase that can act to relieve the block. We took a genetic approach to identify possible phosphatases that can impact Sec4p function. We examined both the PP1 and PP2A phosphatases which account for >90% of all serine/threonine dephosphorylation reactions [30], [31], [32], [33]. We examined the impact of single phosphatase mutations and double mutations in combination with *sec4-8* to determine if these genes may be acting in a common pathway [23], [34], [35].

The PP1 phosphatase Glc7p has been described as playing an important role in cell growth, cell cycle progression and trafficking [36], [37], [38]. We reasoned that if a phosphatase would be positively required to reverse the effect of phosphorylated Sec4p, then removal or diminishment of its activity would compromise Sec4p function. This would lead to synthetic lethality between *sec4* mutants and mutant phosphatase alleles. *GLC7* is an essential gene and we tested two different mutant *glc7* alleles. Neither of these alleles had any significant impact on *sec4-8* in the double mutant (Supplementary Data), suggesting that Glc7p does not influence Sec4p function in vivo.

We next examined the effect of the PP2A phosphatase. PP2A is a heterotrimeric enzyme comprised of three subunits, the structural subunit (A), the catalytic subunit (C), and a single regulatory subunit that dictates the substrate selectivity of the enzyme [39]. Regulatory subunits determine the substrate specificity, (sub)cellular localization and catalytic activity of the PP2A holoenzymes. *S. cerevisiae* has two distinct regulatory subunits encoded by the genes *CDC55* or *RTS1*. PP2A activity is critical for cell survival and deletion of the genes for both regulatory subunits simultaneously results in lethality although cells are still viable with single deletions of either *CDC55* or *RTS1*.

**Figure 3:**
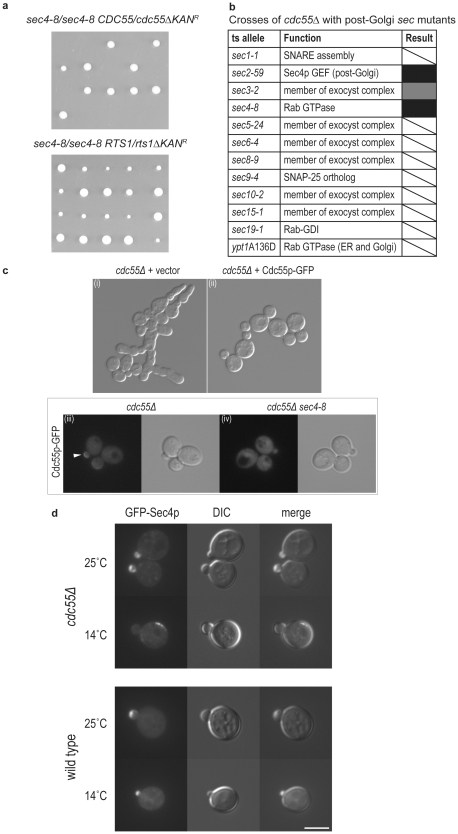
Invovement of PP2A in pathways of exocytosis. **a.** *CDC55* and *RTS1* genetic interactions with *sec4-8*. *sec4-8/sec4-8 CDC55/cdc55Δ*KAN^R^ and *sec4-8/sec4-8 RTS1/rts1Δ*KAN^R^ diploid cells were sporulated and tetrads dissected on YPD media. Five representative tetrad dissections are shown in each case. **b.** Crosses of *cdc55Δ* with post-Golgi *sec* mutants. White boxes with diagonal stripe indicate no interaction, black boxes indicate synthetic lethality at 25°C, and gray boxes indicate that the restrictive temperature of the double mutant was at least 3°C lower than that of the single mutant. **c.** Localization of Cdc55p to the sites of exocytosis is abolished in *sec4-8* mutant cells. The Cdc55p construct is functional when tagged at the COOH-terminus as it can restore normal growth morphology to the *cdc55Δ* cells; (i) *cdc55Δ* cells + vector only (ii) *cdc55Δ* cells + plasmid expressing Cdc55p-GFP under control of the endogenous promoter and terminator (pRC3897A *CDC55*-GFP pRS315). Isogenic (iii) wild type and (iv) *sec4-8* haploid cells containing COOH-terminally tagged Cdc55p-GFP as the only source of Cdc55p were examined for Cdc55p to the sites of exocytosis (white arrow). Cdc55p-GFP is normally distributed throughout the cytosol and also at sites of exocytosis. The enrichment of Cdc55p-GFP at the sites of exocytosis is lost in *sec4-8* cells after a shift to the restrictive temperature when compared to the isogenic wild type cells. **d.** Localization of Sec4p to the sites of exocytosis is abolished in *cdc55Δ* null cells. GFP-Sec4p localization was examined in *cdc55Δ* cells at the permissive (25°C) and restrictive (14°C) conditions in comparison to a wild type control.

To examine genetic interactions beween *sec4* and PP2A, the gene for either *CDC55* or *RTS1* was deleted with a G418 resistance marker in a *sec4-8*/*sec4-8* diploid and the strain was sporulated to generate isogenic haploid progeny, half of which would be expected to contain the deleted PP2A subunit. This analysis is shown in Figure 3A. Half of the progeny from the *sec4-8*/*sec4-8 CDC55/cdc55Δ* diploid (top panel) were inviable and genetic analysis revealed that the surviving progeny were all G418 sensitive. This was not the case for *RTS1* where all haploid cells were viable (bottom panel). These data reveal a synthetic lethal relationship between *sec4-8* and *cdc55Δ*, but not *rts1Δ*. This discrimination between the effects of deletion of either regulatory subunit suggests that the phosphatase PP2A, in the trimeric complex with Cdc55p, but not Rts1p, aids Sec4p function in vivo.

If the regulatory subunit Cdc55p plays a role in aiding Sec4p function, we would also expect that the deletion of this gene would also show a genetic relationship with genes encoding other proteins that closely interact with Sec4p [40]. Using the *CDC55* deletion, we performed crosses with a set of mutants in the post-Golgi trafficking pathway. In addition to *sec4-8*, *cdc55Δ* was synthetically lethal with the Sec4p activator *sec2-59* and was significantly sicker with the exocyst protein *sec3-2* (Figure 3B). These genetic interaction data suggest that dephosphorylation of Sec4p could be mediated by protein phosphatase 2A in a Cdc55p regulatory subunit-specific manner.

### Localization of Cdc55p is dependent upon Sec4p action

To further determine the physiological significance of the interactions between *SEC4* and *CDC55*, we examined the ability of mutations in these genes to influence the cellular localization of the putative partner. The localization of PP2A subunits has been previously established [41]. The two regulatory subunits, Rts1p and Cdc55p, localize independently to different sites within the cell; Rts1p localizes primarily to the nucleus, and Cdc55p localizes to the bud tip and bud neck of cells, suggesting that Cdc55p could regulate a distinct set of substrates involved in polarized growth. Given that our genetic data suggests a positive role of Cdc55p-mediated PP2A function in the phosphorylation cycle of Sec4p, we examined whether Sec4p facilitates the localization of Cdc55p to sites of polarized growth. For these experiments we made use of COOH-terminally tagged Cdc55p-GFP. *cdc55Δ* cells display defects in morphogenesis [42] and these defects could be rescued with the COOH-terminally GFP-tagged CDC55 construct demonstrating that the GFP tag did not interfere with function (Figure 3C (i) and (ii)). The localization of Cdc55p-GFP, expressed as the sole cellular copy of *CDC55* was examined in either wild type or *sec4-8* cells. In wild type cells, Cdc55p-GFP is present throughout the cytoplasm and also enriched at sites of polarized growth (bud tip of small budded cells, and bud neck of cells approaching cell division) in approximately 50% of cells examined at room temperature. After a temperature shift to 32°C for 30 min, the number of wild type cells displaying characteristic localization of Cdc55p-GFP remained the same, however the relative intensity at the sites of polarized growth appeared to improve, perhaps reflecting an overall increase in the rate of vesicle delivery to sites of polarized growth. Conversely, in *sec4-8* cells, a marked decrease in the localization of Cdc55p-GFP was observed at room temperature when compared to the isogenic control strain, and was abolished with a 30 min shift to the restrictive temperature of 32°C (Figure 3C (iii) and (iv)), suggesting that the localization of Cdc55p-GFP follows active secretion regulated by Sec4p. Disruption of Cdc55p function also results in diffused localization of Sec4p, when *cdc55Δ* cells are shifted to the restrictive temperature for 1 hour, Sec4p is no longer localized to the bud tip of small budded cells (Fig. 3D). These results show that Cdc55p and Sec4p can mutually influence each other's localization and suggest that the Cdc55p-containing PP2A phosphatase complex plays a role in regulating phosphorylated Sec4p.

## Discussion

Global analysis of the *S. cerevisiae* proteome identified the Rab GTPase Sec4p as a multi-site phosphoprotein with sites of phosphorylation on residues S8, S11, S201, S204 [2], [5]. In this study, we have made use of mutations at these sites to examine the biological consequences of Sec4p phosphorylation. Replacement of the identified phosphorylation sites with glutamic or aspartic acid residues eliminates Sec4p functionality, while introduction of alanine residues at these positions does not impact function. In this context, we interpret glutamic and aspartic acid residues to be acting as phosphomimetics [43], [44]. This interpretation is reinforced by the finding that substitutions of similar sized but neutral residues have no effect in these positions, and also by the fact that mutations at a selective subset of these sites gave rise to conditional *sec4* mutants that accumulate vesicles at the restrictive temperature. These data suggest that phosphorylation is not necessary for Sec4p function but rather suggest that it serves to sensitize Sec4p function.

**Figure 4:**
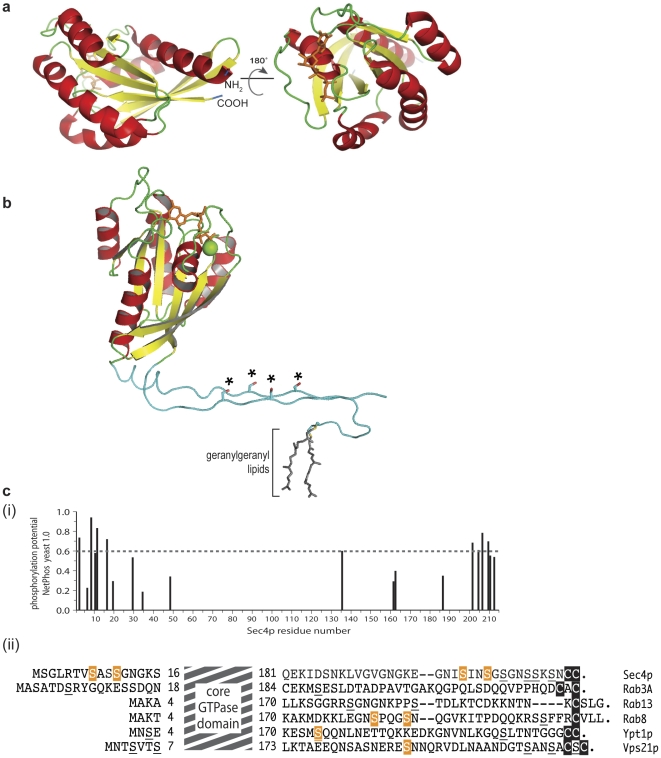
Structural and bioinformatic predictions for the sites of Sec4p phosphorylation. **a.** Diagram showing the relative positions of the boundary residues of the core GTPase domain relative to the nucleotide binding pocket. Structural determinations for the core GTPase domain of Sec4p [87] reveal that the boundary residues of the core GTPase domain are physically located closely in three-dimensional space and are on the opposite face of the protein to the nucleotide binding cleft and switch regions. The terminal residues are shown in blue (the NH_2_ residue at position 19 and the COOH-terminal residue of the core domain at position 187) with the nucleotide in orange. Cartoon representation generated using MacPyMOL (DeLano Scientific) with PDB accession number 1G17. **b.** Hypothetical model showing the peptide extensions from the core GTPase domain of Sec4p. The ribbon diagram shows the relative size of the modeled NH_2_ and COOH tails (colored turquoise) for Sec4p including the geranylated di-cysteine motif at the extreme COOH-terminus. The positions of S8, S11, S201, and S204 are indicated with an asterisk. The tails are modeled using MacPyMOL (DeLano Scientific), extending from the core GTPase domain with approximately the same trajectory as Ypt1p for which a structure is available that includes these regions of the protein [28], [88]. **c.** (i) Graphical output of the predictive algorithm NetPhos Yeast [62], [63] (http://www.cbs.dtu.dk/services/NetPhosYeast/) for the primary sequence of Sec4p showing a clustering of sites with high probability (p>0.6, dotted grey line), at the protein termini, and includes the residues verified experimentally and analyzed in this study. (ii) Alignment of peptide extensions at the NH_2_- and COOH-termini reveals phosphorylated serines and other potentially phosphorylated residues in these termini are also apparent in the exocytic Rab proteins Rab13, Rab8 and Rab3A. Also shown are the equivalent regions of the yeast Rab proteins Ypt1p and Vps21p that contain phosphorylated serine residues. Shaded in orange are serine residues for which phosphorylation has been experimentally determined, underlined are residues that score with p>0.6 as potential phosphorylation sites with NetPhos 2.0 (http://www.cbs.dtu.dk/services/NetPhos/). Note the length of the peptide extensions of these Rab GTPases compared to other Ras superfamily members such as Cdc42Hs with a total protein length of 191 residues, or K-Ras (protein length 188 residues).

To understand the mechanistic consequences of Sec4p phosphorylation we examined the ability of the protein with phosphomimetic substitutions in the positions of phosphorylated serines to undergo nucleotide binding and hydrolysis. We did not observe any significant impact of these mutations on the GTPase cycle, either intrinsically or under the influence of its known direct regulators Dss4p, Sec2p and Gyp1p. This conclusion is supported by structural considerations: the peptide regions containing the phosphorylated residues are on the opposite side of the protein to the nucleotide binding cleft (Figure 4A) and are not part of the molecule that engages the activators Sec2p and Dss4p [45], [46], [47]. In contrast, our data suggest that one mechanistic consequence of in vivo Sec4p phosphorylation is to block interaction with the exocyst component effector Sec15p as phosphomimetic substitutions in these positions rendered the protein unable to interact with Sec15p but still retained interaction with another regulator, Rab-GDI. Sec15p action is essential for polarized exocytosis and is the only known effector of Sec4p that is necessary for viability, suggesting that the inability of the phosphomimetic Sec4p to provide function is a direct consequence of its lack of interaction with Sec15p. The exact mode of interaction between Sec4p, its effector Sec15p and other Sec15p interacting components, including the polarity establishment protein Bem1p [48], the Sec4p exchange factor Sec2p [49], the unconventional myosin Myo2p [50], [51], [52], [53], the other seven subunits of the exocyst [54], [55] and the other Ras-related small GTPases that bind to the exocyst, remain to be understood. The crystal structure of the exocytic ortholog of Sec4p, Rab3A, in complex with its effector Rabphillin, demonstrates a binding mode made up of non-contiguous regions of Rab3A that includes the extended NH_2_-terminus [56]. In a similar fashion, the extended regions at either or both of the termini of Sec4p may constitute a binding determinant for Sec15p that can be blocked in a regulated manner by phosphorylation. Replacement of the phosphorylated residues with the negatively charged phosphomimetics may lock Sec4p in an inactive state because it can no longer form productive interactions with its effector. When not engaged in effector or other protein-protein interactions, these particular residues will be physically accessible to the action of phosphoregulatory enzymes (Figure 4A and B), being located in unstructured regions that protrude from the core GTPase domain. In support of phosphorylation as a negative regulatory event on Sec4p-mediated trafficking we identified the PP2A phosphatase as a component modulating exocytosis through its Cdc55p, but not the Rts1p regulatory subunit. *CDC55* shows genetic interactions with *sec4-8* and other genes required to facilitate Sec4p function. In addition the localization of Cdc55p is affected in *sec4* mutants and Sec4p is mislocalized in *cdc55Δ* cells. These results suggest the hypothesis that the localization of the Cdc55p regulatory subunit of PP2A may reflect a role in secretion and could potentially directly facilitate Sec4p action via the removal of inhibitory phosphates.

The presence of extensions of around 5–30 residues that project from the NH_2_- and COOH-termini of the core GTPase domain [8], [57] is a common feature of Rab proteins that differentiates them from their Ras superfamily relatives. These short peptide regions have long been recognized to contain specificity determinants for individual Rab GTPases [58], [59], [60], [61]. Residues in these regions of Sec4p and mammalian Rab homologs [8] also score highly in bioinformatic prediction alogrithms of phosphorylatable residues [62], [63] (Figure 4C (i)) as they are both intrinsically unstructured and physically accessible. Of particular note is the structural determination of Rab6 in complex with its effector GCC185 which suggests a model where the long hypervariable region is extremely exposed as the Rab helps tether the GCC185-Arl1 complex to the membrane surface [64]. It is tempting to speculate that this feature could be exploited to regulate the timing and extent of Rab-mediated tethering.

The peptides containing the phosphorylated residues in the termini of Sec4p are not present in crystal structures available for Sec4p. However, these structures reveal that the beginning and end of the Sec4p core domain, residues 18 and 187 respectively, are situated in close physical proximity on the same side of the molecule (Fig. 4A and 4B). Mutations of the individual sites of the phosphorylated serines hint at a complex use of these multiple sites for signal integration. Although residues in the Sec4p NH_2_-terminal extension appear to be the most important for determining Sec15p interactions, the impact of these residues can be modulated by phosphorylation on the COOH-terminal residues. Such multi-site phosphorylation could be the product of either a single kinase or by multiple kinases in a hierarchical fashion [65]. Certainly the clustering of phosphorylated serines and the physical proximity of the extreme NH_2_- and COOH-termini could make this system well suited for the action of kinases that require prior phosphorylation at a residue in the vicinity of their own phosphorylation site. Such a mechanism may also be applicable to other Rab proteins, for instance, the Rab proteins Ypt1p and Vps21p are phosphorylated in the COOH-terminal region (Fig. 4C (ii)). In mammalian cells, Rab9, Rab7A and Rab8A are also known to be phosphorylated in the tail region [66] and the COOH-terminal tail of Rab4 is a substrate for p34*^cdc2^* kinase [66], [67], [68]. Other Rab proteins have also reported to be phosphorylated although the biological consequences of phosphorylation are not known [69], [70].

The potential interplay of several closely apposed phosphorylation sites may serve to increase the complexity, sensitivity, and/or cooperativity of the system [71]. The biological strategy of such a mode of regulation can be straightforwardly rationalized in terms of the coordination of traffic with other cellular events. Exocytosis in *S. cerevisiae* is closely coupled to the cell cycle and morphogenesis pathways. As such, Sec4p function is expected to be under control of the same signaling and checkpoint pathways that have been described for the molecular and morphological progressions of cell growth. This may be analogous to a role for Rab1 and Rab4 phosphorylation in conjuction with cell cycle regulation that has previously been suggested [72].

In metazoa, phosphorylation cascades controlling cell cycle progression have been well established to influence membrane trafficking events, particularly at the Golgi apparatus [73], [74], [75], [76], [77], [78], [79], but also in endocytosis [80], [81]. There is also an accumulating body of knowledge that describes the effects of particular kinases on the secretory and endocytic systems [80], [81], [82], [83], [84], [85], [86]. Clearly phosphorylation, as a universal means of coordinating diverse physiological outputs, plays diverse roles in membrane traffic. We suggest that our study reveals a view of the Rab GTPase Sec4p that incorporates a regulatory phosphorylation mode into the existing cycles of nucleotide binding/hydrolysis and translocation between membranes and cytosol. Future studies are needed to address the timing and spatial contexts of the phosphorylation events as well as the signal transduction network(s) which impinge on these events.

## Supporting Information

Figure S1 The functionality of Sec4p to be tagged at its NH_2_-terminus was examined by comparing (1) an untagged *SEC4* construct with constructs expression *SEC4* fused to (2) GFP, (3) MBP, (4) GST, and (5) vector alone (no *SEC4*), as a negative control. Constructs were transformed into *SEC4Δ* cells, and resulting transformants were struck onto media with and without 5-FOA and incubated at 25°C for 3 days.
(PDF)

Figure S2 (A) Interactions with Guanine Nucleotides. (A) Dissociation kinetics of mant-GDP from recombinant Sec4p proteins containing either phosphomimetic (ASP) or alanine (ALA) substitutions in the positions of the phosphorylated serines, in comparison to Sec4p wild type (WT). Recombinant proteins were produced from either pET15b or pGEX4-T vectors. Plasmids were expressed in E. coli BL21-DE3 and grown to OD600 ∼0.4 prior to induction with 0.5 mM IPTG for 4 h at 37°C. Cultures were harvested by centrifugation, resuspended in lysis buffer (50 mM Tris pH 8.0, 200 mM NaCl, 10 mM MgCl2, 1 mM PMSF, 1 mM benzamidine-HCl, 1 µg/ml pepstatin A) and sonicated on ice. Total lysates were clarified by centrifugation at 28,000×g for 15 min. Recombinant proteins were purified on affinity resin according the manufacturer's instructions. Purified proteins were concentrated and stored in 20 mM Tris pH 8.0, 50 mM NaCl, 100 mM KCl, 40% glycerol. Protein concentrations were determined with a standard Bradford assay. Recombinant His_6_-Sec4p proteins (500 nM) were loaded with mant-GDP and fluorescence was measured at 447 nm as relative fluorescence units (RFU) over time after addition of excess unlabeled nucleotide. Single-phase exponential decay kinetics were fit using Prism (v4.0). No significant differences could be observed for rate constants between wild type Sec4p and Sec4p mutants (∼0.0019 sec^−1^). (B) Nucleotide Exchange Assays with Sec4p Exchange Factors Sec2p and Dss4p. His_6_-Sec4p phosphomutants (500 nM) were pre-loaded with mant-GDP before the addition of either unlabeled GDP (50 µM) alone, or in combination with Sec2p amino acids 1–182 (0.15 µM) or Dss4p (1 µM) in buffer 50 mM Hepes pH 8.0, 200 mM NaCl, 1 mM EDTA, 1 mM DTT, 5 mM MgCl_2_, 0.1% Lubrol. Sec2p nucleotide exchange assays were performed at 17°C, reactions with Dss4p were carried out at 30°C. (C) Gyp1p-stimulated GTP Hydrolysis of recombinant Sec4p proteins containing either phosphomimetic or alanine substitutions in the positions of the phosphorylated serines. Sec4p proteins (3 mg) were pre-loaded with GTP (3 mM) and incubated for 1 hr at room temperature before being passed over a gel filtration column, to remove excess unbound nucleotide. Assays were conducted using 20 µM loaded GTPase and initiated with His_6_-Gyp1p (2 µM) or buffer alone. A standard curve for inorganic phosphate release was generated using a phosphate standard in place of GTPase. Gyp1p catalyzed rates of GTP hydrolysis from Sec4p or phosphomutants were nearly identical (0.027 mol P_i_ released/mol Sec4p/min for wild type protein and values of 0.0298 and 0.0289 for the Sec4p^ALA^ and Sec4p^ASP^ mutants respectively). Recombinant Gyp1 was a kind gift of D. Lambright. Inorganic phosphate was measured using the EnzChek Assay Kit (Molecular Probes). This assay measures the generation of inorganic phosphate by its transfer to the substrate 2-amino-6-mercapto-7-methylpurine riboside (MESG) by purine nucleoside phosphorylase (PNP) resulting in an absorbance shirt from 330 nm to 360 nm. (D) GDI Inhibition Assays. The ability of Rab-GDI to prevent the loss of GDP from Sec4p was evaluated using Sec4p and Sec4p^ASP^ (1 µM), preloaded with mant-GDP (1 µM). Reactions were initiated by the addition of unlabeled GDP (100 µM) in the presence or absence of 5 molar excess of recombinant Rab-GDI and monitored for the loss of fluorescence at 447 nm in buffer 50 mM Hepes pH 8.0, 200 mM NaCl, 1 mM EDTA, 1 mM DTT, 5 mM MgCl_2_, 0.1% Lubrol. Rab-GDI retards the rate of GDP loss from Rab proteins. In vitro, no discrimination could be observed between the recombinant Sec4p versions tested.
(PDF)

Figure S3 Genetic interactions between Protein phosphatase 1 and *sec4-8*. RCY2805 (*sec4-8 GLC7*Δ [pRS316 GLC7]) was transformed with *glc7^ts^* mutants, *glc7-10* or *glc7-13*, wildtype *GLC7* or vector (pRS315) before being frogged to either YPD or 5-FOA containing media. RCY2757, an isogenic control strain lacking *sec4-8*, was used to compare cell growth with the single *glc7^ts^* alleles. *sec4-8* shows no genetic interactions with either *glc7^ts^* mutant.
(PDF)

Figure S4 Graphic summarizing the URA3 plasmid shuffle system. This system begins with a cell line where the genomic copy of *SEC4* is deleted and viability maintained with an episomal copy of wild type *SEC4* in a plasmid containing the *URA3* marker. The construct to be tested is transformed into this strain using a second selectable marker (*LEU2*). These transformants are plated on 5-FOA containing media. The product of *URA3* acts on 5-FOA to generate a toxic product that kills the cell (Boeke, J. D., LaCroute, F. and Fink, G. R. (1984) Mol. Gen. Genet. 197, 345). The cell can survive by eliminating the *URA3* containing plasmid, which renders the cell dependent on the alternative construct as the only cellular source of Sec4p function (sec4*). If this construct (a mutated version of SEC4) can provide Sec4p function, colonies are able to form on the 5-FOA-containing media. In contrast, lethality will be observed if the construct is unable to provide Sec4p function.
(PDF)

Table S1 *Saccharomyces cerevisiae* strains used in this study.
(PDF)
