# Supplementary figures and images for: Phosphorylation Provides a Negative Mode of Regulation for the Yeast Rab GTPase Sec4p
Source: PLoS One. 2011 Sep 12;6(9):e24332. doi: 10.1371/journal.pone.0024332 (PMC3171412; doi:10.1371/journal.pone.0024332)

Supplemental Figure 1

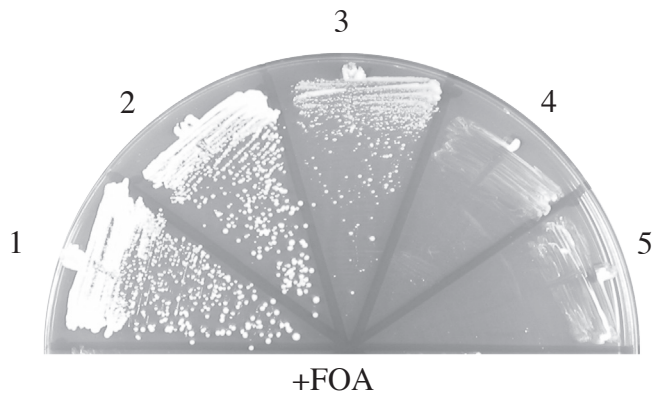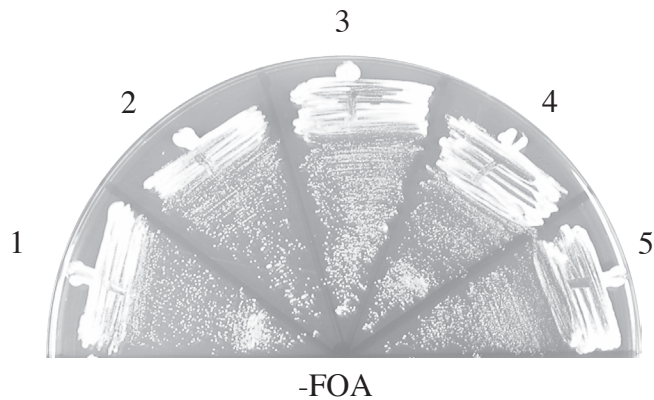

Supplement: Figure S1 — The functionality of Sec4p to be tagged at its NH2-terminus was examined by comparing (1) an untagged SEC4 construct with constructs expression SEC4 fused to (2) GFP, (3) MBP, (4) GST, and (5) vector alone (no SEC4), as a negative control. Constructs were transformed into SEC4Δ cells, and resulting transformants were struck onto media with and without 5-FOA and incubated at 25°C for 3 days. (PDF) [file pone.0024332.s001.pdf]

Supplemental Figure 2

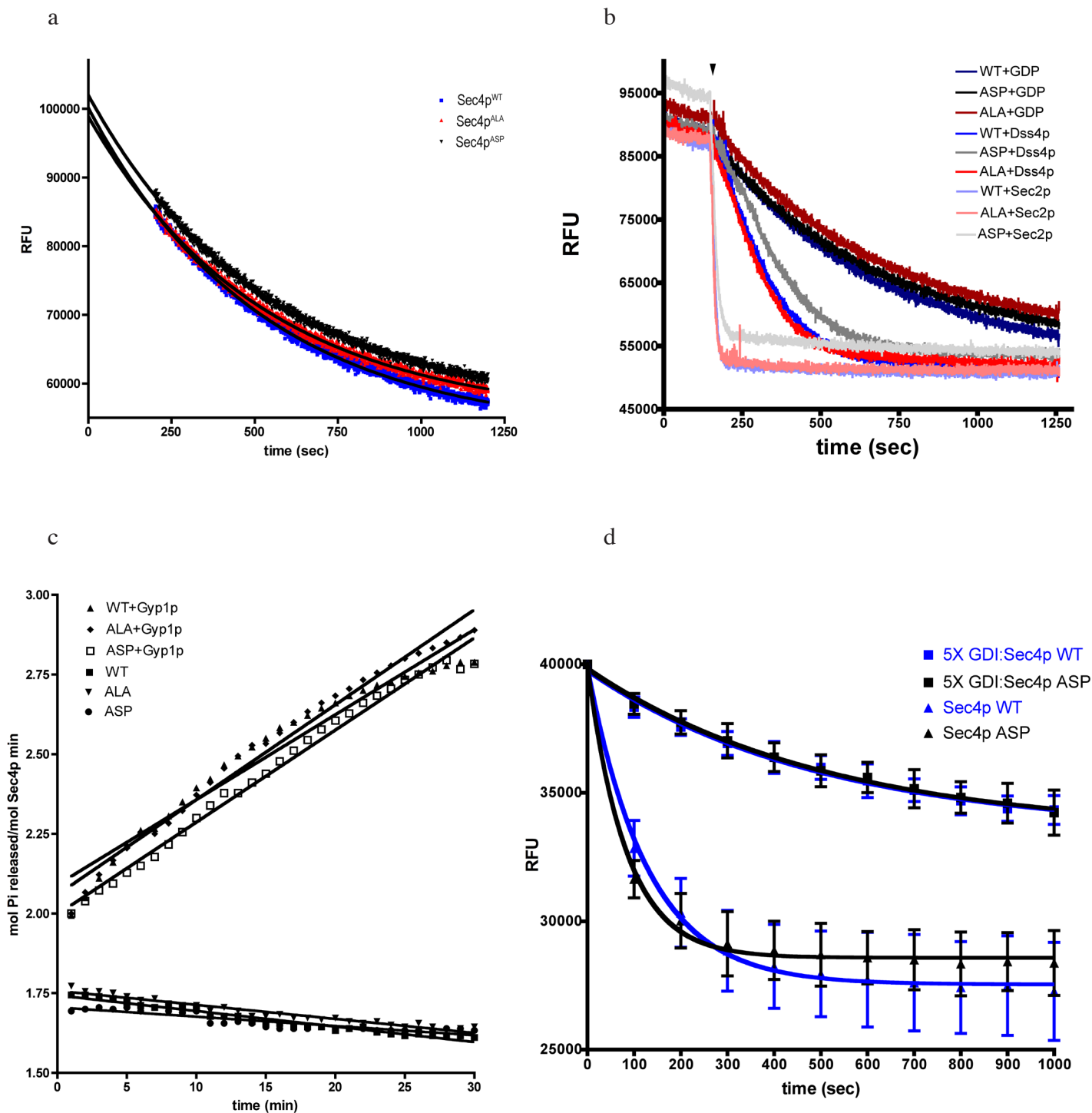

Supplement: Figure S2 — (A) Interactions with Guanine Nucleotides. (A) Dissociation kinetics of mant-GDP from recombinant Sec4p proteins containing either phosphomimetic (ASP) or alanine (ALA) substitutions in the positions of the phosphorylated serines, in comparison to Sec4p wild type (WT). Recombinant proteins were produced from either pET15b or pGEX4-T vectors. Plasmids were expressed in E. coli BL21-DE3 and grown to OD600 ∼0.4 prior to induction with 0.5 mM IPTG for 4 h at 37°C. Cultures were harvested by centrifugation, resuspended in lysis buffer (50 mM Tris pH 8.0, 200 mM NaCl, 10 mM MgCl2, 1 mM PMSF, 1 mM benzamidine-HCl, 1 µg/ml pepstatin A) and sonicated on ice. Total lysates were clarified by centrifugation at 28,000×g for 15 min. Recombinant proteins were purified on affinity resin according the manufacturer's instructions. Purified proteins were concentrated and stored in 20 mM Tris pH 8.0, 50 mM NaCl, 100 mM KCl, 40% glycerol. Protein concentrations were determined with a standard Bradford assay. Recombinant His6-Sec4p proteins (500 nM) were loaded with mant-GDP and fluorescence was measured at 447 nm as relative fluorescence units (RFU) over time after addition of excess unlabeled nucleotide. Single-phase exponential decay kinetics were fit using Prism (v4.0). No significant differences could be observed for rate constants between wild type Sec4p and Sec4p mutants (∼0.0019 sec−1). (B) Nucleotide Exchange Assays with Sec4p Exchange Factors Sec2p and Dss4p. His6-Sec4p phosphomutants (500 nM) were pre-loaded with mant-GDP before the addition of either unlabeled GDP (50 µM) alone, or in combination with Sec2p amino acids 1–182 (0.15 µM) or Dss4p (1 µM) in buffer 50 mM Hepes pH 8.0, 200 mM NaCl, 1 mM EDTA, 1 mM DTT, 5 mM MgCl2, 0.1% Lubrol. Sec2p nucleotide exchange assays were performed at 17°C, reactions with Dss4p were carried out at 30°C. (C) Gyp1p-stimulated GTP Hydrolysis of recombinant Sec4p proteins containing either phosphomimetic or alanine substitutions in the positio [file pone.0024332.s002.pdf]

# Supplemental Figure 3

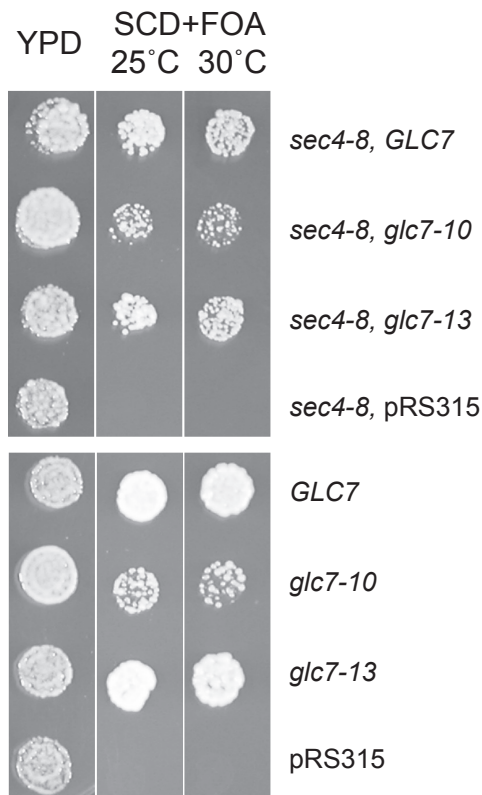

Supplement: Figure S3 — Genetic interactions between Protein phosphatase 1 and sec4-8. RCY2805 (sec4-8 GLC7Δ [pRS316 GLC7]) was transformed with glc7ts mutants, glc7-10 or glc7-13, wildtype GLC7 or vector (pRS315) before being frogged to either YPD or 5-FOA containing media. RCY2757, an isogenic control strain lacking sec4-8, was used to compare cell growth with the single glc7ts alleles. sec4-8 shows no genetic interactions with either glc7ts mutant. (PDF) [file pone.0024332.s003.pdf]

Supplemental Figure 4

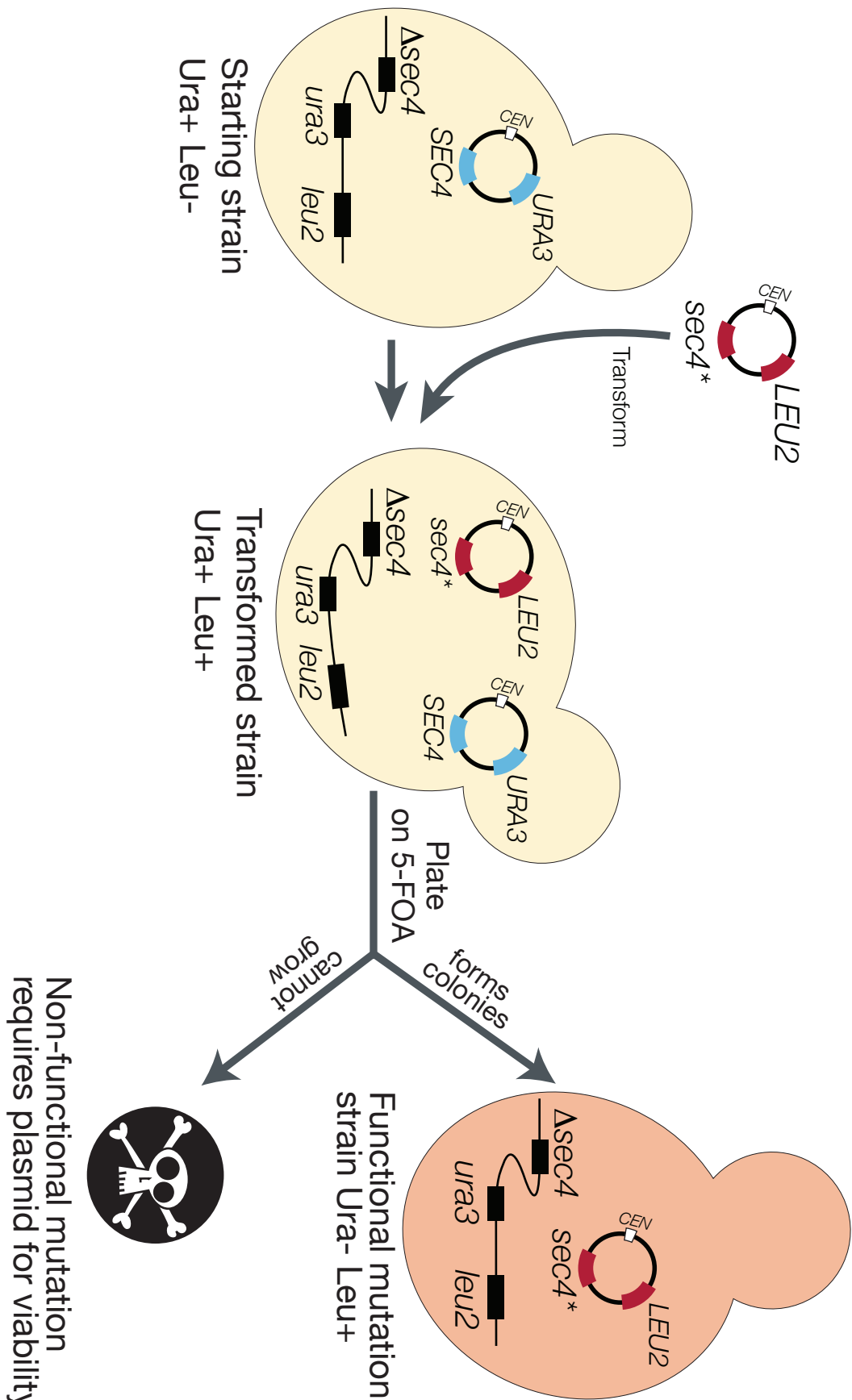

Supplement: Figure S4 — Graphic summarizing the URA3 plasmid shuffle system. This system begins with a cell line where the genomic copy of SEC4 is deleted and viability maintained with an episomal copy of wild type SEC4 in a plasmid containing the URA3 marker. The construct to be tested is transformed into this strain using a second selectable marker (LEU2). These transformants are plated on 5-FOA containing media. The product of URA3 acts on 5-FOA to generate a toxic product that kills the cell (Boeke, J. D., LaCroute, F. and Fink, G. R. (1984) Mol. Gen. Genet. 197, 345). The cell can survive by eliminating the URA3 containing plasmid, which renders the cell dependent on the alternative construct as the only cellular source of Sec4p function (sec4*). If this construct (a mutated version of SEC4) can provide Sec4p function, colonies are able to form on the 5-FOA-containing media. In contrast, lethality will be observed if the construct is unable to provide Sec4p function. (PDF) [file pone.0024332.s004.pdf]
