# Supplementary material for: Phosphorylation Provides a Negative Mode of Regulation for the Yeast Rab GTPase Sec4p
Source: PLoS One. 2011 Sep 12;6(9):e24332. doi: 10.1371/journal.pone.0024332 (PMC3171412; doi:10.1371/journal.pone.0024332)
Supplement: Table S1 — Saccharomyces cerevisiae strains used in this study. (PDF) [file pone.0024332.s005.pdf]

Table S1.
Saccharomyces cerevisiae strains used in this study

Strain	Genotype	Source
NY774	*MAT***a** *ura3-52 leu2-3,112 sec4-8*	Novick lab
Y190	*MAT***a** *gal4Δ gal80Δ trp1-901 ade2-201 ura3-52 leu2-3,112 URA3::GAL10 →LacZ, LYS2::GAL10 →HIS3 cyh*^{R}	Elledge lab
RCY1507	*MATα ura3-52 leu2-3,112 his3Δ200 SEC4ΔHIS3* [YCP50 *SEC4*]	Calero et al.
RCY2802	*MAT***a** *ura3 leu2 his3Δ0 sec4-8*	This study
RCY2805	*MAT***a** *ura3 leu2 lys2Δ0 sec4-8 GLC7ΔKAN*^{R} [pRS316 *GLC7*]	This study
RCY3049	*MATα ura3-52 leu2-3,112 sec3-2*	This study
RCY3217A	*MAT***a**/*α ura3/ura3 leu2/leu2 his3Δ0/his3Δ0 sec4-8/sec4-8*	This study
RCY3316	*MAT***a**/*α ura3/ura3 leu2/leu2 his3Δ0/his3Δ0 sec4-8/sec4-8 CDC55/ cdc55ΔKAN*^{R}	This study
RCY3350	*MAT***a** *ura3Δ0 leu2Δ0 his3Δ0 lys2Δ0 cdc55ΔKAN*^{R}	This study
RCY3424B	*MAT***a** *ura3-52 leu2-3,112 cdc55ΔKAN*^{R} *sec4-8*	This study
RCY3499A	*MAT***a** *ura3-52 leu2-3,112 cdc55ΔKAN*^{R}	This study
RCY3501	*MATα ura3Δ0 leu2Δ0 his3Δ0 lys2Δ0 met15Δ0 rts1ΔKAN*^{R}	This study
RCY3464C	*MATα ura3-52, leu2-3,112 his3Δ200 cdc55ΔKAN*^{R} *SEC4ΔHIS3* [YCP50 *SEC4*]	This study
RCY3682A	*MATα ura3-52 leu2-3,112 his3Δ0 sec15-1 SEC4ΔHIS3* [YCP50 *SEC4*]	This study
RCY4338	*MAT***a**/*α ura3/ura3 leu2/leu2 his3Δ0/his3Δ0 sec4-8/sec4-8 RTS1/rts1ΔKAN*^{R}	This study
